# Plant-derived extracellular vesicles: a synergetic combination of a drug delivery system and a source of natural bioactive compounds

**DOI:** 10.1007/s13346-024-01698-4

**Published:** 2024-08-28

**Authors:** Mattia D. Langellotto, Giovanna Rassu, Carla Serri, Sara Demartis, Paolo Giunchedi, Elisabetta Gavini

**Affiliations:** 1 PhD Program in Biomedical Sciences - Neuroscience, Department of Biomedical Sciences, University of Sassari, Sassari, 07100 Italy; 2 Department of Medicine, Surgery and Pharmacy, University of Sassari, Via Muroni 23/a, Sassari, 07100 Italy

**Keywords:** Plant-derived extracellular vesicles, Exosome, Bioactive, Drug delivery system, Nanovesicles, Nanoparticles

## Abstract

Exosomes are extracellular nanovesicles secreted by all cell types and have been studied to understand and treat many human diseases. Exosomes are involved in numerous physiological and pathological processes, intercellular communication, and the transfer of substances. Over the years, several studies have explored mammalian-derived exosomes for therapeutic and diagnostic uses. Only recently have plant-derived extracellular vesicles (EVs) attracted attention for their ability to overcome many defects associated with using mammalian-derived extracellular vesicles, such as safety and scale-up issues. The ease of large-scale production, low toxicity, low immunogenicity, efficient cellular uptake, high biocompatibility, and high stability of these nanovesicles make them attractive for drug delivery systems. In addition, their native contents of proteins, miRNAs and secondary metabolites could be exploited for pharmaceutical applications in combination with other drugs. The present review intends to provide adequate tools for studying and developing drug delivery systems based on plant-derived EVs. Therefore, indications concerning extraction methods, characterisation, and drug loading will be offered. Their biological composition and content will also be reported. Finally, the current applications of these systems as nanocarriers for pharmacologically active substances will be shown.

**Graphical Abstract:**

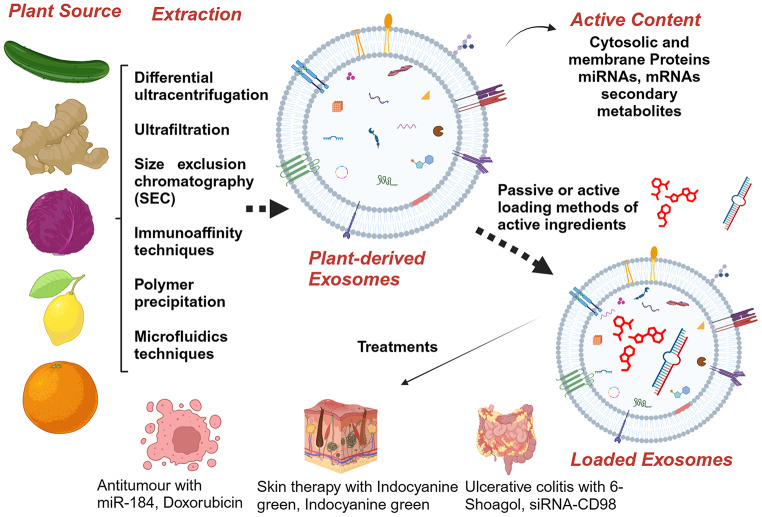

## Introduction

Exosomes are extracellular vesicles (EVs) secreted by many cell types, such as animals, vegetals and bacteria. Plant-derived exosomes have a phospholipid bilayer architecture with a diameter ranging between 30 and 150 nm [1]. These vesicles originate from membrane invagination and fuse in early endosomes. The endosomes then develop into multivesicular bodies (MVBs) as various cytosolic components accumulate near the small membrane curvatures of the endosome and become swallowed inside, forming exosome precursors. The MVBs merge with the membrane and release vesicles into the extracellular space [2, 3]. Plant-derived exosomes possess varied contents, including proteins, lipids, DNA, mRNAs, and microRNAs [4]; the composition varies depending on the origin and extraction method. Previous studies concluded that they act by removing waste from cells [5], but further research has shown their essential role in cell-to-cell communication during physiological and pathological processes [6]. Moreover, exosomes are capable of interspecies and interkingdom communication, especially the delivery of different bioactive compounds [7, 8]. The physiochemical arrangement of exosomes allows them, once ingested, to carry their cargo without being degraded by lysosomes [9]. However, the complete separation of the exosome component from the extracellular vesicles is difficult to achieve, leading to the common occurrence of a mixture of different subpopulations of exosomes. This challenges the interpretation of research findings and underscores the need for caution when referring to specific exosome populations. For these reasons, it is often more accurate to refer to general EVs rather than exosomes [10]. Mammalian-derived exosomes were the first and most extensively studied type of EVs in the past. This is mainly because of their specific targeting ability and involvement in different pathological states. They are also considered nanovectors that can be used to deliver various therapeutics, such as for lung cancer treatment [11]. Exosomes derived from human mesenchymal stem cells also exhibit potential as regenerative therapeutic agents [12]. However, concerns have been raised about the immunogenicity and safety of mammalian-derived exosomes. Mammalian-derived exosomes participate in a large number of physiological and pathological processes in the body. Among their functions, they are involved in the removal of waste material, such as substances that can promote tumour development, exogenous RNA, and pathogenic agents. Additionally, human-derived exosomes do not grant an appropriate scalable manufacturing process [13]. The purification steps for mammalian exosomes also pose obstacles to separating them from the supernatant of tissue cultures [14]. Due to the complexity of the biological fluid, contamination often occurs with non-vesicular components such as lipoprotein and nucleoprotein complexes. Other types of extracellular vesicles can also be found, which may be of comparable size and density. The presence of other active materials and the variability within the subpopulation of EVs make a reproducible separation process and quantification of the content quite challenging [15].Therefore, plant-derived EVs are becoming more attractive because they can overcome these defects. Plants can meet the challenge of disposing of an adequate quantity of safe and low-cost sources of exosomes. Compared with other manufactured nanoparticles, plant-derived EVs are safe, non-toxic, and biocompatible [16]. In addition, they have better absorption and distribution, resist in the digestive tract, easily pass through biological membranes and are more stable as they tend to have a negative surface zeta potential [1, 2, 16–18]. These EVs can also withstand digestion by intestinal and gastric enzymes [19]. Given their composition, plant-derived EVs have various pharmacological activities [6, 20]. For instance, grapefruit-derived EVs have been shown to immunomodulate the inflammatory response in the intestine [19]. Exosomes derived from *Allium tuberosum* have been shown to alleviate microglial inflammation [21]. Taken together, these qualities and properties make plant-derived EVs appealing nanocarriers for drug delivery. Their innate therapeutic components and ability to transport and protect cargo could be exploited synergistically. However, synthetic nanovesicles, such as liposomes, have already been developed for drug-delivery purposes. Several studies have demonstrated their ability to pass through biological barriers, improve drug distribution and reach specific targets [1, 22]. Nevertheless, liposomes have a strict protocol involving numerous and onerous chemical steps that complicate their preparation and modification [23]. Furthermore, synthetic nanoparticles present immunogenicity and cytotoxicity issues [16]. The PEGylation process on liposomes, which aims to reduce their clearance by the mononuclear phagocyte system, actually reduces the ability of the complex to reach and interact with the target. Secondly, an immune response often develops with the production of antibodies directed against PEG [14]. Considering all this information, this review aims to illustrate the main characteristics of plant-derived EVs and their use as a nanotechnological tool for drug delivery systems, considering the work carried out in recent years. In particular, this analysis intends to emphasise their potential as drug carriers in synergy with their innate therapeutic abilities.

### EVs extraction from plant sources

Plants are rich sources of material for the production of EVs, ranging from roots to leaves and from fruits to seeds, and every part of these materials can be used for this purpose. Each part must be evaluated concerning particle size and quantity and different bioactive content, as distinct species or even other parts of the same plant may have diverse concentrations of EVs. Another aspect that should be considered is EVs’ naturally occurring pharmaceutical endeavour: protein, RNAs and other chemicals found in plants and, therefore, in exosomes must be investigated for their pharmacological activities.Several strategies are available for isolating exosomes from their plant matrix, which differ in equipment, time and resource consumption and complexity (Table 1).

**Table 1:** Summary of isolation methods with their benefits and drawbacks

Method	Advantages	Disadvantages
Differential ultracentrifugation	Simple to perform, sufficiently pure exosomes, can withstand large volumes.	Expensive machinery, long time to perform, low yield, possible coprecipitation with other contaminants and membrane disruption.
Ultrafiltration	Higher efficiency than ultracentrifugation, no special equipment is needed, shorter time to be performed.	Filter clogging, some exosomes may be trapped, and the high pressure could damage the exosome.
Size exclusion chromatography (SEC)	Simple and economical, does not damage the membrane, high reproducibility.	It demands specific equipment, is hard to scale, and is lengthy to perform.
Immunoaffinity techniques	Extremely pure product and elevated specificity.	Poor ligands are available; it is expensive and has challenging operation conditions.
Polymer precipitation	Simple and cost-effective, stable yield and can manage large volume.	Low purity and exosomes may aggregate with protein.
Microfluidics techniques	Fast and high sensitivity, kit ready-to-use.	It has a low yield and is more suitable for diagnostic purposes. Difficult standardisation.

**Fig. 1:**
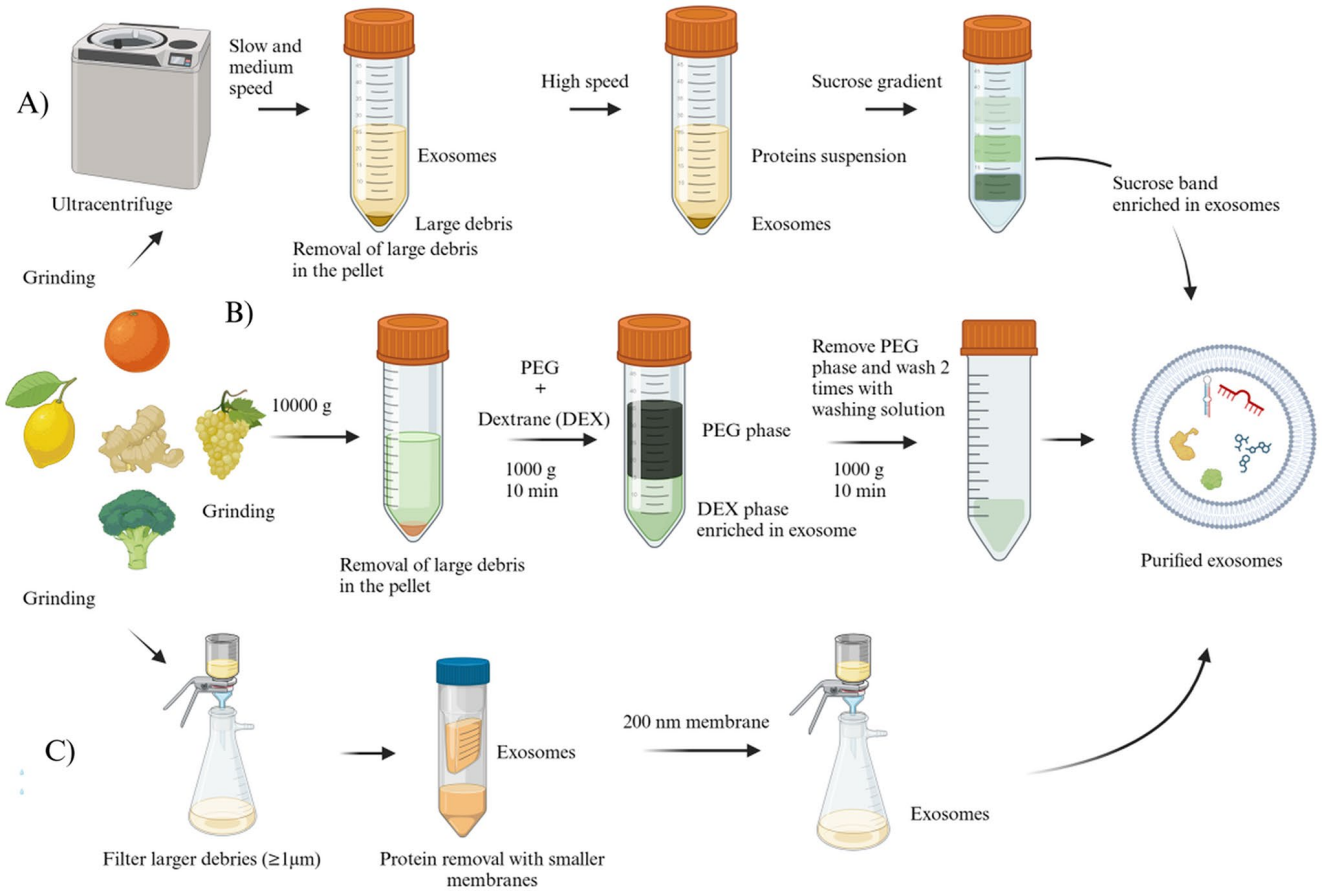
A schematic illustration of different common methodologies for exosome extraction from natural sources. (**A**) Differential ultracentrifugation followed by sucrose gradient purification. (**B**) The peculiar development of a polymer precipitation approach named isolation through an aqueous two-phase system. (**C**) An ultrafiltration process could be combined with differential ultracentrifugation (created with https://biorender.com)

The gold standard for exosomes isolation is the differential ultracentrifugation method and purification on a sucrose gradient (Fig. 1A). This procedure is based on differences in the density and size of the particles to be separated [23].

A juice is obtained from the carefully washed and squeezed or blended organic fruit. After that, a two-stage centrifugation is performed, starting at low speed followed by a high-speed revolution, to remove primarily the large debris and the larger cell fragment. Final ultracentrifugation produces a pellet that is suspended in PBS and transferred to a 30% sucrose cushion. This last purification step makes it possible to effectively remove the last small contaminant, such as proteins, from the exosome that floats in the cushion. The EVs recovered from the cushion were washed and ultracentrifuged, and ultimately, the precipitate was collected [1, 8]. Li and coworkers observed that ginger-derived exosomes purified with a sucrose cushion are neater, more spherical and have fewer aggregated particles [24]. Ultracentrifugation is simple to perform and inexpensive since it does not require solvents or substances. However, this process is too time-consuming and requires expensive machinery. The obtained exosomes are only moderately pure with a low yield due to the high force, which results in aggregation of the particles, coprecipitation with some proteins and disruption of the exosome [25, 26]. To overcome this disadvantage, ultrafiltration (Fig. 1C) is used: it is a size-based technique involving membrane filters and pressure, resulting in faster separation and does not necessitate special equipment. The efficiency of this method is greater than that of ultracentrifugation, and the time required is shorter [27]. Despite the benefits, there are disadvantages to consider: the filter may clog, and some particles may become trapped, compromising the membrane’s durability by lowering the separation efficiency. Additionally, pressure could deform and damage the lipid membrane [23, 28]. Size exclusion chromatography (SEC) is a simple and economical method. Working with gravity does not affect the integrity of the exosomes and results in great reproducibility; however, specific equipment is needed, its scalability is difficult, and it takes a long time to perform [29]. Ultrafiltration and SEC are used in combination with other techniques, such as ultracentrifugation, to increase the purity of the product [26]. Immunoaffinity techniques are based on the presence of different types of protein receptors on the membrane surface. This technique makes it possible to create a specific antibody that can selectively bind specific proteins expressed on the lipid bilayer surface when attached to a solid matrix. Highly pure exosomes are obtained in low yield since only particular subtypes of exosomes are recognised [29]. An application of this technique is immunomagnetic beads: it uses magnetic beads to separate plant-derived vesicles under the influence of a magnetic field. This system recognises the surface proteins of plant exosomes. The applied magnetic field is controllable and is capable of isolating all exosomes or selective isoforms of exosomes [30]. Since immunoaffinity is a young technique, only a few ligands are available, and more selective ones must be developed. Moreover, this pricy method is not always suitable because of its practicality and difficult operational conditions [31]. Precipitation methods include the use of polymers and can manage a large volume of starting material [31]. Kalarikka and colleagues used 10% polyethylene glycol (PEG), a highly hydrophilic polymer that entraps exosomes by modifying their solubility. This technique is easy to perform, cost-effective and does not affect the quality of the exosomes. However, due to vesicle aggregation, the process could result in co-isolation with other EVs or other cellular components [32]. This could be partially avoided with preliminary preparation of the sample. Still, additional steps prolong the procedure time, and without a selective method of isolation, the purity of the exosomes is affected [28]. Kim and coworkers reported a separation method that consists of the repartition of exosomes and contaminants in an aqueous two-phase. The system is formed by PEG and dextran (Fig. 1B). Exosomes mainly interact with the dextran phase due to their surface characteristics, allowing their separation and even purification. This approach is a more rapid and affordable process and does not require any specific apparatus, such as an ultracentrifuge. Nevertheless, the presence of dextran in the final product may be problematic due to its cell toxicity. Moreover, it could interfere with several analytical methodologies, such as western blotting and increase the viscosity of solutions [33, 34]. Recent progress has been made with microfluidics techniques, which use a plethora of principles, such as size-based filtration using sieves or immunoaffinity methods. These procedures are simple to use, fast and affordable. Low yield and insufficient standardisation make these techniques unsuitable for drug delivery purposes but more amenable for diagnostic uses [29]. Abraham and colleagues attempted a different and new extraction procedure: a sequence of high-pressure homogenisation cycles was applied to cucumber juice previously milled with an Ultra-Turrax homogeniser, leading to the formation of a plant crystal fraction. This method indirectly revealed that exosomes are formed after the milling process. Still, it was not possible to extract and purify them with conventional methodologies such as size exclusion chromatography and gradient ultracentrifugation [35].

### Characterisation

Transmission electron microscopy (TEM) analysis was used to assess the integrity and morphology of the isolated EVs [26]. Atomic force microscopy (AFM) and cryo-transmission electron microscopy (cryo-TEM) help visualise the shape and dimension of these EVs. It is also important to perform particle size analysis via dynamic light scattering (DLS) or nanoparticle tracking analysis (NTA) [8, 36]. DLS is useful for monodisperse particles, for which NTA can be performed on polydisperse samples since it tracks singFle particles [37]. NTA is still used to measure the zeta potential because it is important since it defines the tendency to aggregate, which affects the stability of the nanoparticles [38]. Zeta potential was shown to influence transport efficiency; indeed, the cellular uptake of grapefruit exosomes was greater than that of tomato-derived exosomes, which are larger and have a more negative zeta potential [37]. Recent studies have focused on the identification of protein markers present on the membrane surface of the EVs, which can be analysed by western blotting and flow cytometry [2]. Zhang and colleagues have used a GFP-AtPEN1 protein marker to label *Nicotiana benthamiana* EVs; PEN1 is a plasma membrane protein involved in the regulation of membrane fusion [39]. Despite this, there are currently few plant-specific markers, whereas those of mammalian EVs are more abundant and studied. Future research would focus on identifying specific markers of plant EVs for their better characterisation and for a deeper understanding of the biological processes in which they are involved. To characterise the protein amount and profile, HPLC coupled with mass spectrometry could be used [40, 41] as well as gel electrophoresis and ELISA [42]. Raimondo and colleagues used GeLC-MS/MS and LC‒MS/MS to analyse proteomic profiles [8]. The protein concentration is often used to assess the number of exosomes [43]. RNAs are detected through several methodologies, such as PCR, microarray analysis and next-generation sequencing techniques [16]. Lipidomic analysis can be performed using a triple quadrupole mass spectrometer [40].

### Biochemical composition

**Fig. 2:**
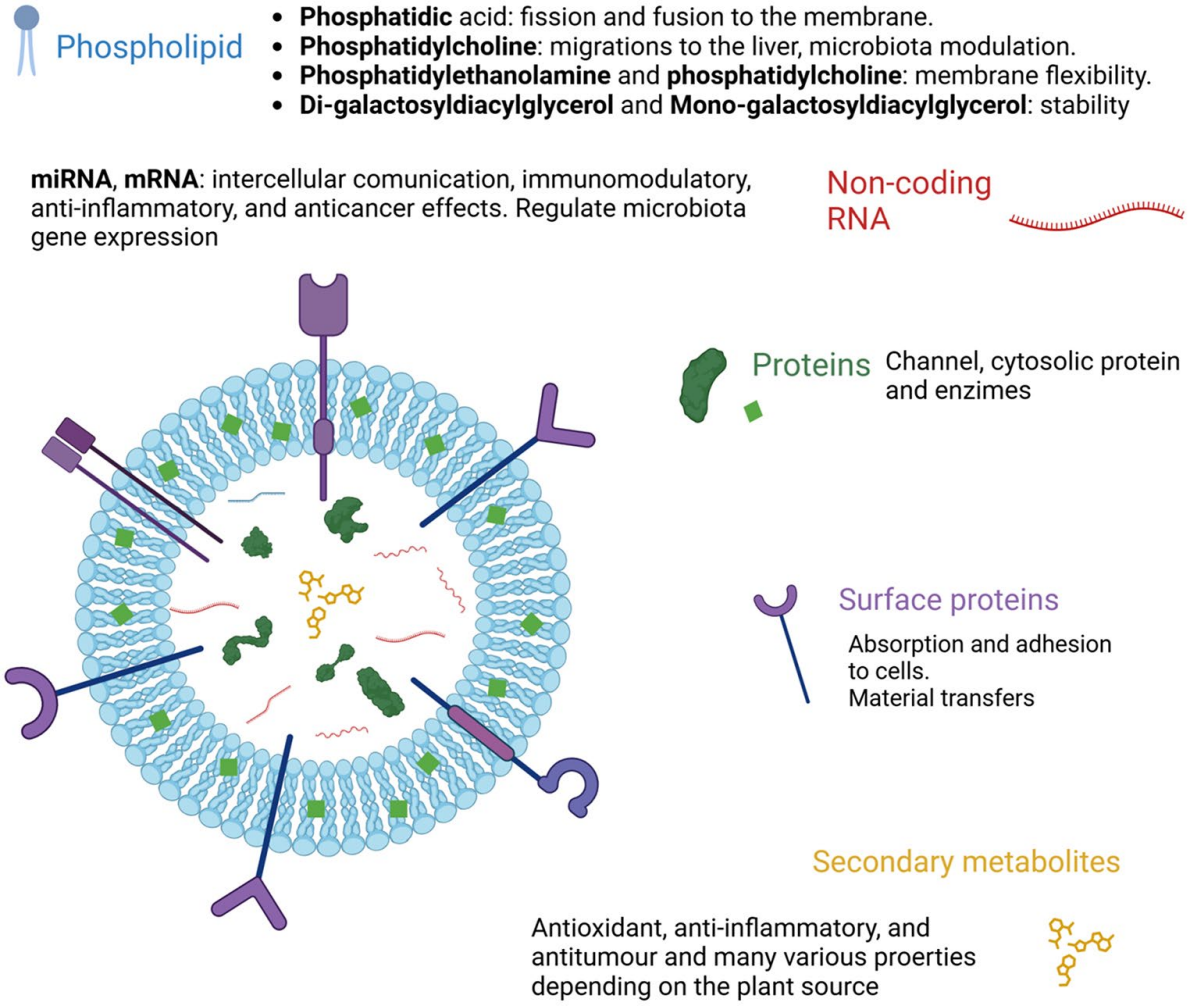
Schematic structural composition of plant-derived exosomes

EVs’ structure, arrangement and internal content may influence their pharmacokinetics and pharmacological activity. Therefore, appropriate composition characterisation is also necessary for understanding its activity [2]. Plant-derived exosomes have a varied internal content that defines their peculiar characteristics (Fig. 2). Lipids are essential for vesicle stability and content protection from gastric digestion [37]. Once administered, the lipid composition can alter the distribution and adsorption of exosomes by intestinal cells. Phosphatidic acid (PA) is important for the control of fission and fusion at the membrane; it appears to act by inducing a rearrangement of the cytoskeleton [44, 45]; PA seems to favour the residence in the intestine [46, 47]. The exosome formation is facilitated by PA, promoting membrane curvature, and recruiting the appropriate cytosolic proteins. PA is prevalent in ginger-derived exosomes and is involved in the taken up by *Lactobacillus rhamnosus* increasing its number in the intestine [47]. Phosphatidylcholine has proved to be involved in the migration of plant-derived exosomes from the gut to the liver [4]. Furthermore, as they can be absorbed by the intestinal microbiota, it has been found that they can modulate the composition and location of intestinal bacteria [47]. Since the lipidic compositions may vary between different types of plants, even their bacterial target may change [47]. Polyunsaturated fats such as phosphatidylethanolamine and phosphatidylcholine make the membrane more flexible; this facilitates their endocytosis since the process demands them to be deformable [4]. Cholesterol, which is present in mammalian-derived exosomes as well as in some liposomes and provides rigidity and stability, is completely absent from plant-derived exosome membranes [48]. Glycolipids such as di-galactosyldiacylglycerol and mono-galactosyldiacylglycerol are involved in membrane stability during lyophilisation [41]. However, the composition is variable; ginger exosomes are mainly enriched in phosphatidic acid and digalactosyldiacylglycerol [41]. Instead, exosomes from grapefruit are mainly composed of phosphatidylethanolamine, phosphatidylcholine, and phosphatidylinositol [19]. Among the substances present in plant-derived exosomes, some of the most attractive are a wide range of noncoding RNAs [49]; these RNAs are known for their function, regulating many physiological processes and taking part in the defence system against pathogens [46]. Among them, mRNAs and microRNAs play an important role in intercellular communication. Xiao and colleagues have analysed microRNAs from 11 edible fruits and vegetables. They revealed their significant role in interspecies and intercellular communication, which has an important role in the post-transcriptional regulation of proteins, either promoting their transcription or favouring their elimination [46, 50]. They have also predicted microRNA human gene targets and revealed possible immunomodulatory, anti-inflammatory, and anticancer effects [50]. It was demonstrated that miRNAs can model the gut microbiota regulating bacterial gene expression, which has been shown to be beneficial in treating colitis in mice [47]. Proteins contained inside plant-derived exosomes are mainly of cytosolic origin and differ from species to species. Channel proteins, such as aquaporin and chloride channels, or cytosolic proteins, like proteolytic enzymes and actin were primarily detected [41]. The protein amount is lower compared to mammalian-derived EVs [46]. An exception was found in lemon-derived exosomes, where the protein level detected was higher than in other plant species, sharing 56.7% with mammalian exosomes [48]. Their functions are various and could change when the plant is harvested during a pathogen contagion or under stress conditions [51]. Certain surface proteins have been indicated to have a role in cellular absorption; their depletion from surfaces has reduced the exosomes assimilation in liver cancer cells [52]. Tetraspanins, integrins and other surface proteins are responsible for adhesions to target cells, enabling the process of material transfer [14]. Plants are renowned for containing a multitude of secondary metabolites that are well known to possess many therapeutic properties. Ginger is renewed for containing 6-gingerol and 6-shogaol, which have antioxidant, anti-inflammatory, and antitumour properties [40]. Vitamin C was found in lemon and strawberry-derived exosomes and naringenin in grapefruit [42], which have antioxidant effects. These molecules may be exploited to boost the effectiveness of a drug loaded in the plant exosome. A meta-analysis that evaluated the application of plant-derived EVs for cancer treatments, showed a general lack of toxicity and safety in their use following oral administration [53]. This strengthens the possibility of their safe use as a drug delivery system.

## Plant EVs as a drug delivery system

Drug delivery systems make it possible to overcome all the problems associated with the administration of substances. For instance, increasing their circulation, reducing their degradation, overcoming side effects, and reaching specific targets, resulting in improved efficacy of the active ingredient (AI). The exigency to develop safe and effective drug delivery systems has pushed the scientific community to investigate many different solutions. Mammalian-derived exosomes have been assessed in recent years because of their properties as natural carriers with intrinsic specific targeting ability. Although many concerns have been expressed about their immunogenicity and cytotoxicity [54], which could be problematic for clinical translation. The need to dispose of an adequate and affordable source of nanoparticles has favoured those of plant origin, as they can overcome these limitations [26]. The following sections report attempts to load plant EVs with active ingredients or models of active ingredients. They are divided into methods to load non-nucleotide substances and ways to load nucleic acids such as siRNA and miRNA.

### Methods for drug loading

**Fig. 3:**
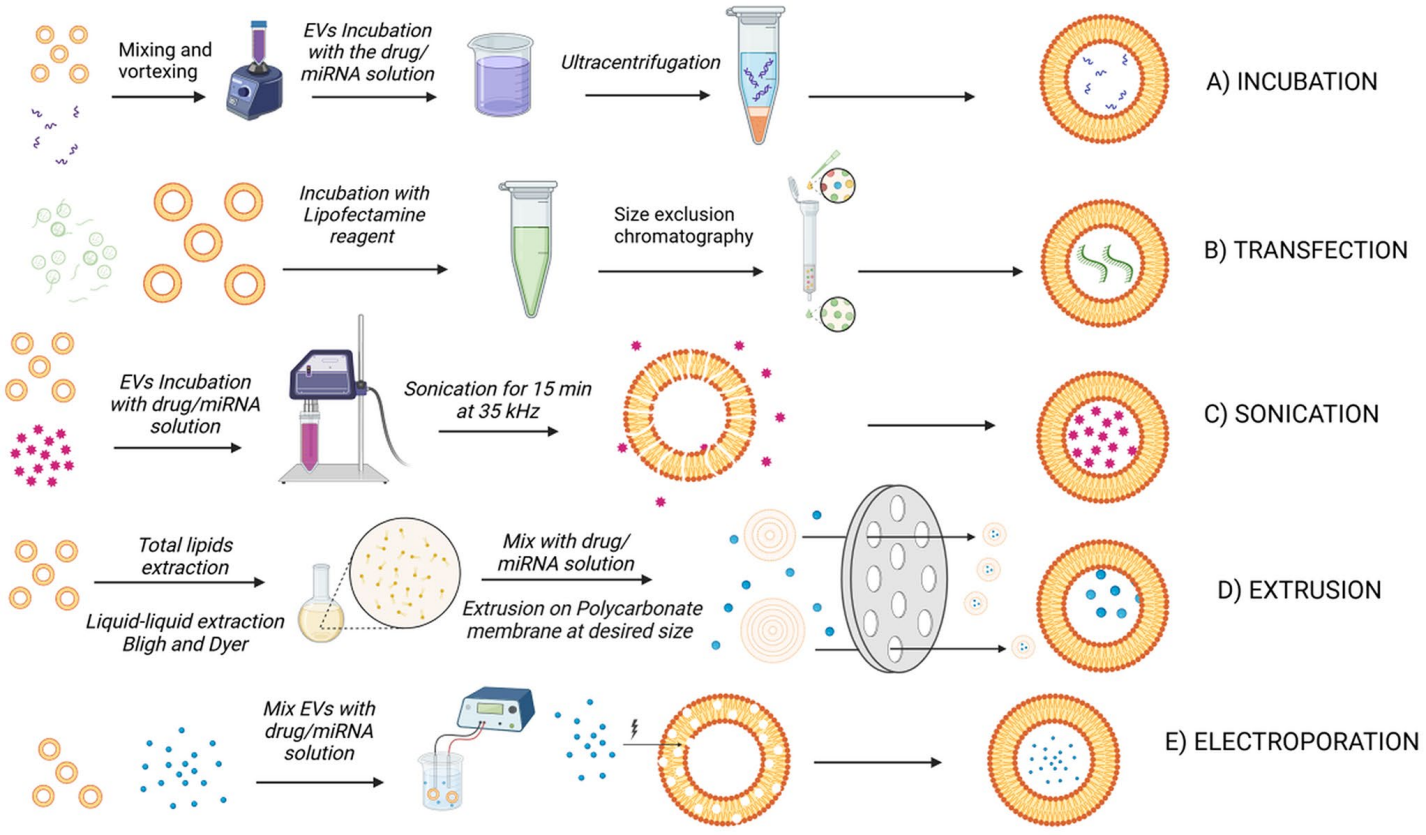
Common drug loading methods for plant-derived EVs

Due to their size and composition, plant-derived EVs are suitable as nanocarriers for small molecules. Plant-derived EVs contain an innate cargo of bioactive components. This load can be used alone or in combination with active therapeutic agents. Several methods are presented below (Fig. 3), many of which are commonly applied for loading other nanoparticle systems. The translation of these techniques from mammalian exosomes or liposomes has been confirmed in several publications. The most common and simple way of loading is by passive methods. This application depends on the ability of hydrophobic molecules to diffuse and interact with the lipidic bilayer [55]. This process consists of incubating the drug-containing solution with the exosomes; the unloaded drug in the solution can be removed by ultrafiltration [35]. You and colleagues incubated a chemotherapy drug, doxorubicin, for 4 h at 37 °C with cabbage-derived EVs. No change in size was detected in EVs after the loading process [26]. Since plant-derived exosome membranes are negatively charged, positive and small molecules can be more easily loaded through the passive method through ionic interactions. Still, negative and neutral-charged drugs have also been loaded within exosomes, which means that their hydrophobicity may prevail over surface electrostatic forces. Lu and a co-worker incubated extracellular vesicles from celery with doxorubicin at 37 °C for 1 h. The system was then precipitated and resuspended after high-speed centrifugation [1]. Abraham and colleagues compared two methods of preparing loaded plant EVs. One method involved mixing the AI with the EVs and using mild vortexing to favour the drug incubation process. The other method involved mixing the AI with cucumber extract before subjecting it to high-pressure homogenisation (HPH). According to the study, the latter method produces plant crystal EVs [35]. To demonstrate the loading efficiency of their method, they compared the results with plant crystal EVs simply incubated with the AI. No dimensional changes were recorded. However, plant crystal EVs produced by adding the AI before HPH had a lesser change in zeta potential than those produced by the incubation method. This result is interpreted as a higher loading rate because there would be less positively charged AI on the surface of the plant crystal EVs, which could reduce the zeta potential. The passive incubation method is effective only for small molecules with favourable physicochemical characteristics. Furthermore, it is more effective for positively charged molecules, as they are more readily drawn to the electronegative charge of EVs [16]. Nevertheless, alternative methods have been explored to enhance the loading efficiency of predominantly negatively charged substances. Multiple cycles of sonication, interspersed with cooling periods, followed by an incubation period at 37 °C are used to enhance the internalisation efficiency of Doxorubicin and Paclitaxel by up to 28% versus 1% with passive incubation and 5% with electroporation [56]. The membrane is momentarily disrupted, allowing the passage of the molecule inside the particles; once sonication ends, the membrane integrity and functionality are restored [57]. Membrane integrity is essential for the functionality of exosomes; therefore, preserving the native properties of plant-derived exosomes via sonication appears not to be the best method for drug loading. Some studies have reported a loss of pharmacological function in exosomes after sonication [58]. Electroporation is largely used for loading other nanovesicles, especially those with hydrophilic molecules; therefore, this methodology is expected to also be effective for plant-derived exosomes [58, 59]. Several parameters must be determined, such as the voltage, pulsation time, and capacity. The membrane is momentarily made porous, enabling the encapsulation of molecules. Both sonication and electroporation can potentially damage the membrane or, in the worst case, lead to a loss of internal contents. The choice of method should always take into account the ultimate goal to be achieved. Gentler loading methods would be more favourable if the aim is to utilise naturally occurring pharmacologically active loading. Plant-derived exosomes can be used directly, without further modification for drug loading, or, occasionally, reconstructed as new nanocarriers following lipid extraction. In this case, it is more appropriate to refer to the new vesicles as EVs mimics, although many use inappropriate terminology such as exosome-like [10]. Lipids from plant-derived EVs are extracted to obtain particles with a precise dimensional range and structure to achieve reproducible results. Bligh and Dyer extraction is one of the most commonly applied methods for this purpose. It is a two-phase liquid-liquid extraction method for obtaining lipids from EVs. Once separated, the lipids are passed through the extruder to achieve the desired size. Exosomes can be mixed with a drug solution during the extrusion to obtain loaded and sized vesicles [60, 61]. Yang and collaborators combined the sonication of the total lipids extracted from ginger and a subsequent extrusion on a liposomes extruder. Sonication helps to assemble the EVs mimics. Following extrusion, the size decreased from 246.4 nm to 180.6 nm, and the zeta potential reduced its negative value yet remained with optimal values for stability. Zhang and colleagues successfully used the same technique to produce ginger EVs mimics containing doxorubicin. The positively charged doxorubicin interacts electrostatically during sonication to be encapsulated within the lipid bilayer. The obtained vesicles have a size of 188 nm and ZP of -15.5 mV with an encapsulation efficiency of 95.9% ± 0.26% [41]. This technique offers high loading efficiency and precise dimensional control. However, the extraction process of the lipid component of EVs may result in the loss of natural molecules and their properties. Freeze-thawing is often used to load drugs into mammalian exosomes; thus, it might be feasible for loading plant-origin EVs. The drug solution is mixed with plant-derived exosomes, rapidly frozen at -80 °C and then thawed at 30 °C in a water bath [59]. Multiple cycles can increase drug loading efficiency, but care must be taken not to overstress the exosomes, risking membrane damage and loss of material. It has also been shown that as the number of cycles increases, size distribution increases and cell uptake efficiency is reduced in a murine melanoma cell line [62]. It was also possible to load proteins inside tomato and grapefruit exosomes; Kilasoniya’s group combined a passive incubation overnight at 4 °C of a suspension of tomato and grapefruit EVs with HSP70 protein and an active loading through sonication at 35 kHz for 15 min. After an extra incubation time, they eliminated the unloaded free protein with a centrifugal filter. Tomato EVs had a bigger particle size (140–170 nm) and more negative zeta potential (-25 mV) compared to grapefruit EVs (86–125 nm and − 10 mV), even if they had better suspension stability. [37]. Only a few attempts were made to load AIs, often preferring to use the natural content of EVs. Each drug with its characteristics may require different solutions to be loaded, frequently, even a combination of several approaches. An additional consideration should be whether or not the ELs need to be unloaded from their contents to achieve a more reproducible system and better control of pharmacological effects.

### Methods for nucleotide loading

Loading nucleotides into exosomes, such as DNA, RNA, siRNA, and miRNA, remains challenging because of their size and negative charge, which can hinder achieving a high yield. One could consider adapting loading methods already used for mammalian extracellular vesicles when it comes to loading drugs. Several attempts to load substances into plant extracellular vesicles have been reported. Cabbage EVs were transfected with miRNAs for 6 h at 37 °C, resulting in high encapsulation efficiency. There was no observed change in size (100 nm) or zeta potential (-14.2 mV) after the loading process. [26]. Umezu and collaborators tested different methods of loading and compared the results. A solution of miRNAs was mixed with Acerola exosomes treated or not treated with CaCl_2_, incubated in an ice bath, and then heat-shocked at 42 °C. They successfully loaded miRNA with all the methods above. The greatest efficiency was found in Acerola exosomes incubated for 30 min in an ice bath without further treatments [22]. Zhang and collaborators extracted total lipids from ginger-derived EVs previously obtained by ultracentrifugation and sucrose gradient centrifugation. After removing the solvent, they mixed the total lipids with siRNAs and sonicated the mixture for 5 min. Finally, the solution was passed through an extruder to achieve the desired size. The resulting size of the EVs mimics was 189.5 nm, slightly smaller than the natural naïve ginger-derived EVs, which were 232.7 nm. The zeta potential remained relatively unchanged after loading with siRNA, shifting from − 18.1 to -18.4 mV. [63]. Interestingly, Pomatto and coworkers used a loading method based on the use of polycationic substances that seem to modify the surface charge of the lipid membrane, enabling the entry of the mRNA. This patent method called cation-based interaction combined with controlled osmotic shock, enables the drug loading to slightly increase the size dimension from 167 ± 10 nm to 227 ± 24 nm after the process. They also compared that technique with passive incubation, noting that the loading was considerably less efficient [64]. In general, it seems that the use of active loading methods or a combination of several methods can increase the loading yield of nucleotides within EVs.

### Storage

More research is needed on the storage of plant-derived exosomes. Currently, only limited information is available, and most results are based on research on mammalian exosomes. Developing a reliable storage method for plant exosomes after extraction and purification is crucial for preventing cargo degradation. Maintaining membrane integrity is essential for their activity. Storage at a temperature below − 70 C° has been seen to be optimal for exosomes. To avoid membrane damage, cryoprotectants such as trehalose, dimethyl sulfoxide or glycerine are used [58]. Storage at temperatures above 25 °C is not recommended [65]. Zhang and colleagues, for instance, reported any morphological or functional changes in ginger-derived exosomes after 25 days of storage at 4 °C [63]. Freeze-drying could be useful for preserving plant-derived exosomes at higher temperatures without affecting their size, morphology or other parameters. Still, it may reduce protein activity, as reported by Noguchi and coworkers, who observed a diminished biological activity of protein saporin after its encapsulation in EVs and freeze-drying [66]. Freeze-dried material is easier to store and can be reconstituted by adding water; the exosomes’ activity and content are retained. However, cryoprotectants like trehalose are recommended since the lipid bilayer can be corrupted during ice crystal formation [67]. A cycle of freezing and thawing reduces the number of exosomes and the protein content. These data suggest that the acidic environment affects exosome stability. Protein degradation is slowed proportionally to decreasing temperatures. However, it has been suggested that exosomes from different sources may respond differently to different processes and thus have variable stability [46]. Plant-derived exosomes have been shown to resist both stomach and intestinal conditions. At the same time, pH interferes with vesicle dimension, increasing and reducing the size of pant exosomes depending on their composition [40, 48]. Kim and colleagues tested the influence of the preservative TMO at -20, 4, 25 and 45 °C compared to that of untreated exosomes extracted from *Dendropanax mellifera* over 4 weeks. They observed no significant change in pH (approximately 5 pH) or in dimensions, which remain between 30 and 200 nm. They also concluded that exosomes treated with TMO at 4 °C were the most stable formulations. Finally, they found that exosomes protected with TMO had significantly better cellular uptake than unprotected exosomes [62].

## Plant-derived EVs loaded with drugs: applications

The vast diversity of plant sources results in a wide variation in their internal content, leading to a wide range of potential applications for these fascinating systems. Several currently available drugs have been loaded into exosomes derived from plants (Table 2) and studied.

**Table 2:** Summary of the most recent drug loading attempts on EVs of plant origin with information regarding the source of extraction, the loaded drug, data concerning the average size and loading efficacy, the application for which they were designed and the loading method used

Source	Drug	Particle size	Loading efficacy	Application	Method	Reference
Acerola	hsa-miR-340	352 ± 180, 245 ± 132, and 340 ± 172 nm	60%	Target gene-suppressing effect in the small intestine	Incubation for 30 min on bath ice	[22]
Aloe	Indocyanine green	138.7 nm and 220 nm	Mass ratio of 3:2 for loading	Noninvasive transdermal administration and skin cancer therapy	n.d.	[73]
Bitter melon	5-Fluorouracil	100–200 nm	n.d.	Oral squamous cell carcinoma	Sonication	[70]
Broccoli	Extracellular miRNAs(ath-miR159a, ath-miR166b-3p, ath-miR319a, ath-miR403–3p and ath-miR159b3p)	Range 35–300 nm, mean size 174.3 ± 5.5 nm	n.d.	Antiproliferative on CaCo-2	Lipofection	[68]
Cabbax	miR-184, Doxorubicin	Average size of 100 nm	Doxorubicin (1 μm) or Cabex (1 × 109 particles mL − 1)	Antitumour	Incubation at 37 °C	[26]
Celery	Doxorubicin	111.8-113.7	87%	Antitumour effects	Incubation at 37 °C	[1]
Cucumber	DiI perchlorate (DiI)	167 ± 3 nm	n.d.	Skin therapy	Incubation + vortexing	[35]
Ginger	Survivin RNA	123.5–124.5 nm	80%	Antitumour	Incubation at 37 °C	[24]
Ginger	siRNA-CD98	189.5 nm	61 ± 8%	Treat ulcerative colitis	Sonication and Extrusion (blight and dyer)	[63]
Ginger	6-Shoagol + M2,M3	200 nm	89.1 ± 2.6%	Ulcerative colitis	Sonication and Extrusion (blight and dyer)	[61]
Ginger	Doxorubicin	188 nm	95.9% ± 0.26%	Colon cancer	Sonication and Extrusion (blight and dyer)	[41]
Grapefruit	Doxorubicin (DOX)-loaded heparin-based nanoparticles	135 ± 5 nm	1 µg (protein amount) of EVs contained 1.01, 1.77, and 1.9 µg of DOX in EV-DN1, EV-DN2, and EV-DN3	For Glioma Therapy	n.d.	[69]
Lemon	Doxorubicin	202.2 ± 6.21 nm	18.84 ± 0.56%	Ovarian cancer	Incubation	[71]
Orange	mRNA-based vaccines	167 ± 10 nm	72 ± 11%	Covid-19	Cation-based interaction combined with controlled osmotic shock	[64]
Tomato and Grapefruit	HSP70 protein	140 ± 13	1.10%	Glioma	Incubation at 4° + Sonication at 35 kHz	[37]

n.d. not available

### Tumour treatment

You and colleagues have successfully loaded doxorubicin inside cabbage-derived exosomes. They have proven that nanoparticles can reach the tumour site, inhibiting the proliferation of colon cancer cells, which, combined with their natural ability to inhibit inflammation and apoptosis, makes these nanoparticles effective as drug delivery systems in anticancer therapy [26]. This study also demonstrated the safety of cabbage exosomes, which not only do not reduce cell viability but appear to stimulate cell proliferation. Another study demonstrated the superior absorption effectiveness and therapeutic activity of doxorubicin-loaded celery-derived exosomes compared to liposomes. In fact, celery EVs loaded with doxorubicin have been shown to reduce the tumour mass in mice better than the liposome-doxorubicin system. In this study, toxicity was examined in mice following intravenous injections. The mice showed no signs of deteriorating health. Blood and organ analysis found no signs of toxicity due to celery EVs. After being injected into the peritoneal cavity of mice, EVs from celery were distributed throughout the peritoneal cavity. When injected intravenously, the EVs were more concentrated in the liver, with a stronger signal than intraperitoneal injection within the first 48 h. This study has demonstrated that EVs can persist for a prolonged period, increasing the likelihood of reaching the intended target site. [1]. The use of a delivery system could also decrease side effects due to the toxicity of an AI, as in the case of doxorubicin administered alone, as well as other drugs [31]. Zhang and colleagues used ginger-derived EVs with doxorubicin as a drug delivery system. They improved the ability of the EVs by functionalising the membrane, including folic acid, since folate receptors are present in many tumour cells. Moreover, they tested the cytotoxicity of ginger-derived EVs with several assays like MTT and electric cell-substrate impedance-sensing. They observed their superior safety compared to a well-proven cationic liposome [41]. Del Pozo-Acebo’s group analysed the native miRNAs of broccoli-derived exosomes and selected five promising candidates for loading into the same vesicles to increase the amount of these miRNAs to a pharmacologically effective dosage. They loaded exosomes by lipofection, which increased the expression level of these miRNAs 600-fold. Research has demonstrated that miRNAs are more resistant to gastric digestion when encapsulated in broccoli extracellular vesicles compared to when they are unencapsulated; they have also reduced at 30% the viability of Caco-2 cells, potentially proving useful in reducing the viability of cancer cells [68]. One study showed that the human recombinant protein HSP70 can be loaded in tomato and grapefruit EVs through a sequence of passive and active loading steps. AlamarBlue cell viability assay showed no toxicity after 48 h incubation in a glioma cell line. Protein HSP70 protects cells from stress-induced damage and can boost the responsiveness of tumour cells to chemotherapy. Moreover, these EVs exhibit antioxidant activity due to their active biomolecules, such as flavonoids, ascorbic acid, and anthocyanins [37]. A fascinating system was developed by Niu and colleagues in which small heparin nanoparticles loaded with doxorubicin with grapefruit-derived exosomes were combined. MTT assay on different cell cultures and histological analysis of mouse organs found no toxicity following intravenous administration of EVs. The amino groups of the exosome membrane lipid bind with the carboxyl group of heparin; this 3-dimensional connection increases the loading efficacy of doxorubicin fourfold. Using heparin nanoparticles increases the circulation time of grapefruit EVs in the system. When EVs stay in circulation longer, they have a better chance of passing the Blood-Brain Barrier and entering the glioma tissue. These EVs are more efficient in recognising and entering glioma tumour cells. The study has demonstrated that the EVs penetrate glioma tissue through receptor-mediated transcytosis and membrane fusion. This ability of grapefruit-derived exosomes to pass through the blood-brain barrier enables them to target the tumour site within the central nervous system, resulting in an antiproliferative effect [69]. A study has demonstrated that EVs derived from bitter melon can amplify the cytotoxic effects of 5-fluorouracil and reduce drug resistance during oral squamous cell carcinoma treatment. This effect is attributed to suppressing the NOD-like receptor family pyrin domain containing 3 (NLRP3) by RNAs present in bitter melon EVs. NLRP3 is a receptor associated with the development of 5-fluorouracil resistance. This study highlights the potential synergy between the natural components of plant EVs and existing drugs, especially when the drugs present distinct pharmacokinetic or pharmacodynamic challenges [70]. The surface of lemon-derived EVs was modified by incorporating heparin-CRGD, a cyclic peptide known for its effectiveness in targeting tumours. After loading the vesicles with doxorubicin through incubation, the heparin-cRGD-modified lemon-derived EVs with doxorubicin exhibited promising results in treating ovarian cancer. Specifically, it demonstrated superior anti-proliferative and anti-tumour effects compared to the free drug, largely attributed to increased cellular uptake. Importantly, this system also showed a good biosafety profile [71].

### Treatment of skin diseases

Abraham and coworkers suggested that plant crystal EVs and classical EVs extracted from cucumber could be used as nanocarriers for transdermal application. The study found that a lipophilic AI was twice as effective at penetrating the skin when applied in combination with EVs than in a buffer solution. This increased efficiency was observed in both the EVs prepared using the classical method, which was assumed to have a 100% loading efficacy, and in plant crystal EVs, which seemed to have only achieved 50% encapsulation [35]. Skin absorption is possible since exosomes can pass through trans- and intercellular pathways via lipid fusion, such as phagocytosis, micropinocytosis and clathrin-mediated endocytosis, as well as through hair follicles [31].

### Treatment of intestinal diseases

According to Umezu et al., it has been observed that miRNAs attach to the surface of acerola extracellular vesicles (EVs) through physical interactions rather than electrical ones, given the negative membrane charge of the nanoparticles. This allows the miRNAs to remain intact and reach the intestinal tract, where they can exert their effects locally or be transported to other areas. Following oral administration, miRNAs loaded in acerola EVs were found in the small intestine and other organs. This indicates that EVs can travel to the liver and spleen before spreading throughout the body. The delivery of complex miRNAs with acerola EVs resulted in a higher concentration of cytosolic miRNAs compared to the administration of free miRNAs, suggesting promising potential for localized action in the intestinal epithelium [22]. Yang and colleagues conducted a study highlighting the effectiveness of administering 6-shoagol loaded within ginger lipid-derived nanoparticles (EVs mimetics) for treating ulcerative colitis. They found that 6-shoagol exhibited improved release kinetics after 24 h compared to the free formulation. Furthermore, the study observed robust internalisation of the 6-shoagol-loaded nanoparticles by macrophages, reaching a maximum uptake after 3 h. The bioavailability of the free and encapsulated drug was also assessed, revealing a significantly higher maximum concentration and retention in the colon following oral administration of 6-shoagol-loaded nanoparticles. These findings indicate a greater efficacy in promoting the healing of intestinal mucosa [61].

### Vaccines

Pomatto and coworkers decided to benefit from this potential and studied exosomes derived from orange juice as a delivery system for RNA-based vaccines. They successfully loaded mRNA that expresses viral proteins of SARS-CoV-2. They showed that these vectors reach the target cell and trigger an immune response by producing antibodies and activating immune cells. They also tested different administration modalities and found that oral and intranasal administration was optimal, without any sign of toxicity for the animal [64].

### Clinical trial

Various EVs derived from plants are currently being investigated in clinical trials for their natural pharmaceutical properties. However, only one trial involves EVs that are loaded explicitly with an AI. This particular clinical trial, which commenced in 2011 and is still ongoing, focuses on generic plant EVs combined with curcumin for treating colorectal cancer [72].

## Conclusion and perspective

Utilising plants as a source of exosomes offers a promising solution to the immunogenic and safety concerns associated with exosomes derived from mammals. Given that plants are consumed daily without adverse effects, they demonstrate exceptional biocompatibility and safety. Unlike synthetic nanoparticles, each component of which must undergo safety assessments, plant-derived EVs are innately non-toxic and do not trigger immunogenic responses due to their natural origins. Furthermore, plant-based materials can potentially resolve the scalability issues linked to the production of synthetic or mammalian-derived EVs. Moreover, synthetic nanoparticles do not possess the intrinsic therapeutic substances found in plant-derived EVs, such as proteins, lipids, and RNAs. These play crucial roles in cell-to-cell communication and gene regulation for numerous physiological and pathological processes. Additionally, bioactive compounds like antioxidants and anti-inflammatory agents, commonly found in plant-derived EVs, are highly valuable for pharmaceutical applications as they can be synergistically employed with exogenous drugs. The potential uses of plant-derived EVs are extensive, ranging from their natural form in delivering supplements and micronutrients to serving as supportive therapies for various conditions, including cancer, inflammatory diseases, and skin disorders. Moreover, they can be explored as natural carriers for therapeutics due to their easy accessibility and cost-effectiveness without the need for chemical solvents for isolation. Each region has the potential to examine its indigenous plant life for various therapeutic uses due to the rich diversity of flora. However, standardising their size and content presents a challenge due to the inherent variability in the characteristics and composition of plant-derived EVs stemming from factors such as the harvest season and the plant’s health. Establishing standardised extraction, characterisation, and storage protocols is essential for achieving consistent results that can be applied to the clinical phase. Given the variable nature of plant sources, a universal method is not feasible. Nevertheless, developing a set of general principles based on the specific characteristics of the plant source could provide a foundational framework for diversifying the types of plants used. Furthermore, in-depth research into plant-derived EVs’ pharmacological and target-delivery properties is essential to define their therapeutic applications precisely. Creating a database of protein content can aid in designing specific markers for characterisation. Additionally, information on nucleic acid and secondary metabolite content could be valuable for virtual screening. To harness the full therapeutic potential of EVs, it is crucial to have a comprehensive understanding of the mechanisms governing their cellular uptake and biodistribution. Familiarity with their natural pharmacodynamics is essential for effectively targeting specific therapeutic sites, such as tumour, intestinal, or central nervous system cells. Consequently, current research is increasingly focused on modifying the surface of EVs to achieve precise targeting. Exploiting this valuable resource could lead to innovative advancements in drug delivery, enabling the development of systems that can interface with the body and boost the efficacy of medications through synergistic interactions with their natural components. This has the potential to facilitate the creation of vaccines, supplements, and treatments for conditions that are currently inadequately addressed by existing therapeutic approaches, managing challenges related to the rapid clearance of active ingredients from the body or their poor solubility in biological environments.

## Data Availability

Not applicable.
